# Supplementary material for: An Online Experiment of NHS Information Framing on Mothers’ Vaccination Intention of Children against COVID-19
Source: Vaccines (Basel). 2022 May 4;10(5):720. doi: 10.3390/vaccines10050720 (PMC9143012; doi:10.3390/vaccines10050720)
Supplement: Supplementary file 1 [file vaccines-10-00720-s001.zip › vaccines-1670980-supplementary.pdf]

Supplementary Materials

Table S1. Descriptive Statistics for DV: Vaccine Confidence Index (VCI) by IV1: risk frame and IV2: disease type.

Descriptives

	Treatment Disease String	Treatment Risk String	VCI
N	Covid	Child	127
		Community	137
	Flu	Child	150
		Community	128
Mean	Covid	Child	18.4
		Community	17.7
	Flu	Child	18.8
		Community	18.2
Std. error mean	Covid	Child	0.259
		Community	0.296
	Flu	Child	0.263
		Community	0.351
95% CI mean lower bound	Covid	Child	17.8
		Community	17.1
	Flu	Child	18.3
		Community	17.5
95% CI mean upper bound	Covid	Child	18.9
		Community	18.3
	Flu	Child	19.3
		Community	18.9
Standard deviation	Covid	Child	2.92
		Community	3.47
	Flu	Child	3.22
		Community	3.97

Table S2. Descriptive Statistics for the DV: 4C (Calculation, Collective Responsibility, Complacency, Confidence) by IV1: risk frame and IV2: disease type.

Descriptives

	Treatment Disease String	Treatment Risk String	4C
N	Covid	Child	127
		Community	137
	Flu	Child	150
		Community	128
Mean	Covid	Child	45.7
		Community	44.5
	Flu	Child	47.6
		Community	45.7
Std. error mean	Covid	Child	0.821
		Community	0.799
	Flu	Child	0.749
		Community	0.916
95% CI mean lower bound	Covid	Child	44.1
		Community	42.9
	Flu	Child	46.2
		Community	43.9
95% CI mean upper bound	Covid	Child	47.3
		Community	46.0
	Flu	Child	49.1
		Community	47.5
Standard deviation	Covid	Child	9.25
		Community	9.35
	Flu	Child	9.17
		Community	10.4
